
JOURNAL INFORMATION
==============================
NLM Title Abbreviation: ScientificWorldJournal
Journal ID: ScientificWorldJournal
NLM Journal ID: 101131163
Journal ID: TSWJ

The Scientific World Journal

ISSN: 2356-6140
EISSN: 1537-744X
Publisher: Wiley

ARTICLE INFORMATION
==============================
PMCID: PMC6083992
DOI: 10.1100/tsw.2001.408
Article ID: 397086
Article version: 1
Subjects: Extended Abstract

Evidence for the Differential Distribution of at Least Two Adrenomedullin Receptor Subtypes in the Rat Brain

Chabot J.-G. 1
Juaneda C. 1
Dumont Y. 1
Fournier A. 2
Quirion R. 1
1 Douglas Hospital Research Center and Department of Psychiatry, McGill University, Verdun, QC, Canada H4H 1R3,
2 INRS-Institut Armand-Frappier, Université du Québec, Pointe-Claire, QC, Canada H9R 1G6,
Publication date: 2001
Electronic publication date: 2001 Oct 9
Volume: 1
First page: 38
Last page: 38
Copyright: Copyright © 2001 J.-G. Chabot et al.
Copyright year: 2001
License: This is an open access article distributed under the Creative Commons Attribution License, which permits unrestricted use, distribution, and reproduction in any medium, provided the original work is properly cited.
License URL: https://creativecommons.org/licenses/by/3.0/

==============================
4th International Meeting on Calcitonin Gene-Related Peptide (CGRP)
*TheScientificWorld* (2001) 1(S1), 38
ISSN 1532-2246; DOI 10.1100/tsw.2001.408

### EVIDENCE FOR THE DIFFERENTIAL DISTRIBUTION OF AT LEAST TWO ADRENOMEDULLIN RECEPTOR SUBTYPES IN THE RAT BRAIN

J.-G. Chabot[1], C. Juaneda[1], Y. Dumont[1], A. Fournier[2], and R. Quirion[1]
[1]Douglas Hospital Research Center and Department of Psychiatry, McGill University, Verdun, QC, Canada, H4H 1R3; [2]INRS-Institut Armand-Frappier, Université du Québec, Pointe-Claire, QC, Canada, H9R 1G6

We recently reported that specific [$^{125}$I]human Adrenomedullin$_{13-52}$ (hADM$_{13-52}$) binding sites are very discretely distributed in the rat brain[1]. Furthermore, in rat brain membrane homogenates, we suggested the existence of at least two populations of [$^{125}$I]hADM$_{13-52}$ receptor binding sites[2]. In the present study, we investigated the differential distribution of specific [$^{125}$I]hADM$_{13-52}$ sites that are sensitive and insensitive to hADM$_{22-52}$ in the rat brain. Adjacent coronal rat brain sections were incubated with 35 pM [$^{125}$I]hADM$_{13-52}$ in the presence of increasing concentrations of hADM$_{22-52}$. As previously reported, specific [$^{125}$I]hADM$_{13-52}$ sites were observed in the choroid and linings of ventricles, basal amygdaloid nucleus, neuronal lobe of the pituitary gland, trigeminal nucleus, and cerebellum. Specific [$^{125}$I]hADM$_{13-52}$ binding is fully inhibited by 1 μM hADM$_{22-52}$ in all these brain regions except in the cerebellum. In fact, hADM$_{22-52}$ was able to inhibit only 70% of specific [$^{125}$I]hADM$_{13-52}$ binding sites in the rat cerebellum. These data suggest for the first time that the rat cerebellum contains at least two populations of ADM receptors, one that is sensitive to hADM$_{22-52}$ while the other is resistant. This characterization of cerebellar [$^{125}$I]hADM$_{13-52}$ / hADM$_{22-52}$ -insensitive sites is currently under investigation.
Supported by grant from the CIHR to RQ.

### REFERENCES

1. Juaneda, C. et al. Eur. J. Pharmacol., in press.
2. Juaneda, C. et al. (2000) Soc. Neurosci. 26, 382.
